# Human biomonitoring to assess exposure to thallium following the contamination of drinking water

**DOI:** 10.1371/journal.pone.0241223

**Published:** 2020-10-29

**Authors:** Maria Cristina Aprea, Daniela Nuvolone, Davide Petri, Fabio Voller, Silvano Bertelloni, Ida Aragona

**Affiliations:** 1 Public Health Laboratory, Department of Prevention, Health Agency of South-East Tuscany, Siena, Italy; 2 Unit of Epidemiology, Regional Health Agency of Tuscany, Florence, Italy; 3 Pediatric Division, Department of Obstetrics, Gynecology and Pediatrics, University Hospital, Pisa, Italy; 4 Department of Prevention, Health Agency of North-West Tuscany, Pisa, Italy; Kaohsiung Medical University, TAIWAN

## Abstract

In 2014, in some parts of the water distribution system of the municipality of Pietrasanta (Tuscany, Italy), thallium (Tl) levels above the recommended limits were measured and some restrictions to water usage for drinking and food preparation were imposed. The study aimed to assess Tl exposure and possible health effects by means of a human biomonitoring survey. In the 2014–2016 time frame, 2154 urine and 254 hair samples were taken from different population groups and from a control group. The levels of Tl found in urine and hair were statistically higher in exposed groups than in controls and compared to the reference values for the general population. Concentrations in urine were significantly associated with the geographical origin of the sample, the consumption of drinking water and food grown in local gardens. A significant association was found between urine and hair. No positive associations were found between the Tl levels in hair or urine and several self-reported symptoms and health effects, except for sleep disturbance. The study indicates that the concentration of Tl in drinking water can be traced by urine analysis. Urine and hair have proven to be biological matrices that can be effectively used for the evaluation of Tl exposure. To date, the study represents the most extensive human biomonitoring campaign for the evaluation of the Tl exposure available at international level.

## Introduction

Biomonitoring allows an integrated assessment of absorption into the organism through all the exposure pathways (respiratory, cutaneous and digestive). For this reason it is applied both in professional field (exposed workers) and in living environments (general population) to assess the impact of environmental pollution. For metals, poorly metabolized, the dosage in biological matrices (mainly in urine) is widely applied [1, 2]. Exposure to thallium (TI) for the general population can occur via air, water and food; however, the levels of Tl in the air and in water are generally very low [3]. Because of its rarity in nature, TI is to date not included in the parameters for monitoring the quality of drinking water required by European and Italian legislation [4]. Small amounts of this metal are released into the air by power plants burning coal, cement factories, metal casting and, more recently, industries employing lasers and superconducting materials such as nuclear magnetic resonance, optical fibers and molecular probes to simulate the biological functions of alkali metals [5]. The greatest exposure occurs upon ingestion of food contaminated with TI, which is found in plant products because it is easily absorbed by plants through the roots [5, 6]. Although fish can absorb TI from water, it is not known whether this ingestion contributes to increased levels of Tl in the body [5]. There is no maximum level for TI in food; the dietary intake in the UK is estimated to be approximately 0.005 mg/day [7]. It is considered that Brassicaceae are likely to be the main source of dietary exposure to Tl in food produced on contaminated land [5–8]. Cigarette smoke is a minor source of TI. People who smoke show levels of Tl in the body slightly higher than non-smokers [9, 10].

If absorbed, TI can affect the nervous system, lungs, heart, liver and kidneys. Hair loss and nerve damage have been observed after exposure to large amounts of Tl. Other effects linked to the consumption of unusually large quantities of TI include nausea and vomiting, followed by organ dysfunction, brain lesions and death [5, 11–16]. However, chronic exposure to low levels of Tl by contamination of drinking water, are rarely reported [17, 18] and population health effects of low-level exposure are little investigated [19–22].

Several studies published in the scientific literature report reference levels of Tl in the urine of the general population not occupationally exposed [9, 10, 19, 20, 23–27]. Similarly the Italian Society of Reference Values (SIVR) [1] reports urinary Tl values in the general population in Italy not substantially different from those reported in international literature. There are a lot fewer studies on the presence of TI in other biological matrices [28–32], including blood, serum and hair.

One recent study reports a case of contamination by TI of the drinking water supply system in Valdicastello Carducci, an outlying district (population of around 1,000 inhabitants) of the town of Pietrasanta (LU, Tuscany, Italy). Briefly, in September 2014, the Department of Earth Sciences, University of Pisa (Italy) reported the presence of significant levels of Tl in some water samples in the village of Valdicastello Carducci [31]. The Local Health Authority and the water supplier carried out an extensive monitoring, that confirmed relevant Tl levels in the drinking water distributed in the village. The source of contamination was immediately identified in a spring located very close to an abandoned mining site, immediately north of Valdicastello Carducci. Despite the contaminated spring being disconnected from the water distribution system, a residual contamination was still detected due to releasing of Tl from the contaminated pipelines. Thus in October 2014 the local authorities imposed restrictions of water uses for drinking and food preparation purposes to Valdicastello’s inhabitants. In November 2014 the same restrictions were extended to the historical centre of Pietrasanta, and in July 2015 to its vicinity (Pollino district), because significant levels of Tl were also measured in drinking water distributed. At the same time short-term measures were adopted. The supplier replaced all the piping of the water supply network in the division of Valdicastello and in some areas of the old town centre (approximately 12 km). In the other areas, new pipe cleaning techniques were experimented with, using high pressure water and CO_**2**_. These interventions led to a significant reduction in the levels of TI in water and therefore also the lifting of the ban on use.

In this context, biomonitoring surveys were conducted to determine the amount of TI in the urine and hair of the population living in the contaminated areas (Valdicastello Carducci, historical centre of Pietrasanata, Pollino) and in a suitably selected control group. The role of other potential risk factors and the prevalence of various symptoms and health effects were investigated.

## Materials and methods

### Study characteristics, approval, information to participants and informed consent

This study is configured as a cross-sectional survey, conducted in the period 2014–2016, which involves the collection of urine and hair samples from a portion of the population with the aim of associating the results of the analysis on the samples with the variables from an ad hoc questionnaire. The study is not configured as a clinical trial and does not foresee a subsequent follow-up intervention.

On December 22^**th**^ 2014, with Resolution no. 1259, the Tuscany Region financed the project and approved a Protocol for the coordination of the competent bodies in the context of carrying out activities and interventions for overcoming thallium contamination of public water in the municipality of Pietrasanta. This protocol provided for the establishment of a working group chaired by the Mayor of Pietrasanta, which involved the participation of all the bodies and subjects competent to implement the programme and a representative of the citizens participate. The actions approved by the protocol included the sampling and analysis of hair and urine, the administration of questionnaires, data processing and the publication of reports and scientific articles.

The study was reviewed and approved by the local ethics committee (Comitato Etico di Area Vasta Nord Ovest Toscana, CEAVNO) on June 4^**th**^, 2015, reg. no. 32799. Many samples were collected before approval from the Ethics committee because at the start of the “emergency”, when significant levels of Tl were found in some water samples and the local authorities imposed restrictions of water uses for drinking and food preparation purposes (the Do Not Drink orders), citizens, on a voluntary basis, or after active invitation provided urines and hair sampling. At the same time, the study project was defined and submitted to the Ethics committee for approval.

All participants gave their informed consent to the study; in the case of minors, informed consent was given by parents or anyone exercising parental responsibility. Failure to sign the informed consent form led to exclusion from the study.

By signing the consent, the participants accepted the collection and delivery of urine and hair samples for the determination of Tl, declared that they had received adequate and clear information regarding the biomonitoring, and that they understood the usefulness and limits of the proposed analyses and any subsequent observational studies, based on the results of the examinations, for epidemiological and prevention purposes, only with respect to professional secrecy and the privacy law. The participants also stated they will immediately inform the Director of the Department of Prevention, Health Agency of North-West Tuscany in case of withdrawal of consent. The consent was also signed by the doctor responsible for collecting the samples.

The information from the Director of the Department of Prevention, Health Agency of North-West Tuscany, provided to the participants at the time of signing the consent, included the process of sample treatment in the following way: 1) each sample, marked with the name and surname of the participant, is recorded and made anonymous with the attribution of a specific identification code; 2) all the anonymized and coded samples are sent to the Public Health Laboratory (Siena, Italy), for the analytical determination of thallium; 3) the Public Health Laboratory (Siena, Italy) transmits the analytical results to the Director Department of Prevention, Health Agency of North-West Tuscany which reserves the right to carry out any subsequent observational studies, based on the results of these tests, processed anonymously and aggregate, for epidemiological and prevention purposes only; 4) each participant is given their analytical report in a strictly confidential form; 5) the report concerning a minor is given to parents or anyone exercising parental responsibility (the same delegated to sign the informed consent).

### Eligibility criteria and population recruitment methods

The sample is made up of male and female participants aged between 0 and 100 years. Exclusion factor was occupational exposure to metals and, in particular, were excluded people who work in foundries, cement factories, the manufacture of jewellery and costume jewellery, thermometers, semiconductors, the laser and glass industries, mining, incineration plants of any type, landfills, collecting and/or processing of waste of any kind.

At the time of delivery of the biological sample, suitably trained personnel administered a specific questionnaire including, in addition to the standard information, all the items deriving from the known sources of exposure to Tl described in the scientific literature and in particular information on education, body mass index, physical activity, smoking habits, the consumption of locally produced food (mode of production with particular regard to the watering), the consumption of water from the aqueduct, the nature of current work activity or work undertaken in the past, diet, various pathologies and symptoms potentially associated with past or ongoing exposure to Tl.

All urine and hair samples were collected in the time interval October 2014 –June 2016.

Urine samples differed on the basis of the time of sample collection, the geographical area, the sample collection method (volunteers and active call by the local health authority) and the main purpose of collection, and were divided into the six groups specified below:

- Group 1: VOLUNTEERS A. The 636 samples that constitute this group (male/female ratio 0.90) were collected on a voluntary basis within two weeks from the Do Not Drink order. The persons lived in Valdicastello Carducci and Pietrasanta old town centre. Samples were collected in October 2014 in Valdicastello Carducci and in November 2014 in Pietrasanta old town centre.
- Group 2: VOLUNTEERS B. The 696 samples that constitute this group (male/female ratio 0.81) were collected on a voluntary basis some time after the end of exposure; 86% of these samples were provided by the Volunteers A, with the addition of other samples from people who had not participated in the first phase of collection. The persons lived in Valdicastello Carducci and Pietrasanta old town centre. This sampling had the purpose of evaluating the level of contamination at a time distance from 1 to more than 4 months from the Do Not Drink order. Samples were collected from November 2014 in Valdicastello Carducci and from December 2014 in Pietrasanta old town centre.
- Group 3: ACTIVE INVITATION PIETRASANTA TOWN CENTRE. The 334 samples that constitute this group (male/female ratio 0.81) were collected by active invitation to the people who had not volunteered. Using the patient registry, written invitation was sent to all local residents (3,498 people) who had not provided a urine sample voluntarily, with the aim of gathering additional participants. These people lived in the old town of Pietrasanta centre and samples were collected several months after the ban in the period May-October 2015.
- Group 4: POLLINO A. The 155 samples of this group (male/female ratio 0.93) were collected after active invitation in July 2015, within two weeks from the day after the drinking water ban was issued.
- Group 5: POLLINO B. This is a group of 118 samples (male/female ratio 0.89) collected after active invitation around 30 days after the end of the exposure in the Pollino district (August-September 2015).
- Group 6: CONTROL GROUP. This consists of 217 volunteers (male/female ratio 1.05). All these persons lived outside the areas of exposure. People who frequently visited the areas of exposure for reasons of work, study or other were excluded. Samples were collected in the time frame February-June 2016.

With regard to hair, all samples were collected in the time interval April 2015 –June 2016. We examined 14 samples from people who live in Valdicastello Carducci (Volunteers April 2015), 138 samples from people who live in Pietrasanta town centre (active invitation May-October 2015), 43 samples from people who live in the Pollino district (active invitation July-September 2015) and 59 samples from persons who live outside the areas of exposure (control group February-June 2016). As with the urine samples, people who frequently visited the areas of exposure for reasons of work, study or other were excluded from the control group.

### Urine and hair sampling and analyses

The analysis of Tl in both urine and hair samples were performed in ICP/MS (Perkin Elmer DRC II) using gallium as internal standard. The methods, developed by the laboratory, were approved by the Italian Accreditation Body (ACCREDIA). The limit of quantification (LoQ) of 0.010 micrograms/L in urine and 0.100 ng/g in hair samples proved to be completely adequate for determining the levels of Tl in the population studied. The expanded uncertainty (probability 95%, coverage factor K = 2) was lower than 20% at all levels of concentration for urine and hair. All the samples were prepared under a laminar flow hood to prevent contamination.

The urine samples were collected in plastic containers without the addition of preservatives and stabilizers and stored in a freezer at a temperature below -20°C. Before the analysis they were diluted with 1% nitric acid. External quality controls (2 samples per month) provided and managed by the Occupational and Environmental Laboratory of Medicine (OELM) ensured the accuracy of analysis results. Urinary creatinine was measured in each urine sample by Jaffe’s colorimetric method [33]. The analytical results were expressed in micrograms/L and in micrograms/g creatinine. Creatinine values were also used to determine whether the spot urine sample was valid and all samples with creatinine concentrations within the World Health Organization guidelines of>0.3 g/L and<3.0 g/L were considered for the statistical analysis [34].

The hair samples (200–400 mg) were taken from the nape of the neck, as close as possible to the scalp, using stainless steel scissors. After collection, they were placed in plastic bags and kept at room temperature. Before the analysis the samples were washed and digested in a microwave oven. Internal quality controls in all the analysis sets ensure the accuracy of the results. The results were expressed in ng/g.

### Statistical analyses

For urine and hair samples a description was given including total sample size, number of outliers and, only for urine, sample size with creatinine concentrations within the WHO guidelines (>0.3 g/L and<3.0 g/L). The values below the LoQ were replaced with the corresponding value of LoQ/2 before the statistical analyses. The outliers were identified using the Grubbs test. The distribution of the measured values of Tl concentration was right-skewed and, in order to obtain a normal distribution, the data were ln-transformed prior to statistical analyses.

Differences of ln-transformed Tl levels among different sample groups were evaluated by parametric test ANOVA. The association between ln-transformed Tl levels and the basic characteristics of the subjects was analysed using the linear regression model (univariate and multivariate) and including all potential predictors, selected a priori, on the basis of the knowledge about the determinants of Tl exposure. The final multivariate model was chosen to provide the greatest value of the adjusted R^**2**^. In order to take into account the association between included variables and urinary creatinine, the models were constructed using Tl urinary levels as the dependent variable and urinary creatinine as a predictor, instead of using it to normalize the urinary Tl concentration, as recommended by Barr et al. [35]. As a sensitivity test, the analyses were also conducted with creatinine-normalized Tl concentrations, instead of including creatinine as a covariate.

Several statistical tests (Pearson, Spearman and Kendall) were used to evaluate the strength of the correlation between Tl levels in urine and hair. Spearman and Kendall correlation tests were used for non-normally distributed data.

Multivariate logistic regression analyses were fitted to test the associations between self-reported symptoms and Tl concentrations adjusting for potential confounders. In these logistic models including Tl levels as independent variables, Tl urinary concentrations were log2 transformed because of the skewed distribution, and to interpret the results as an odds ratio associated with the doubling of Tl levels. Because of the heterogeneity in population recruitment, methods and time frame of urine collection, all regression models were run separately for each group of samples.

## Results

**Fig 1:**
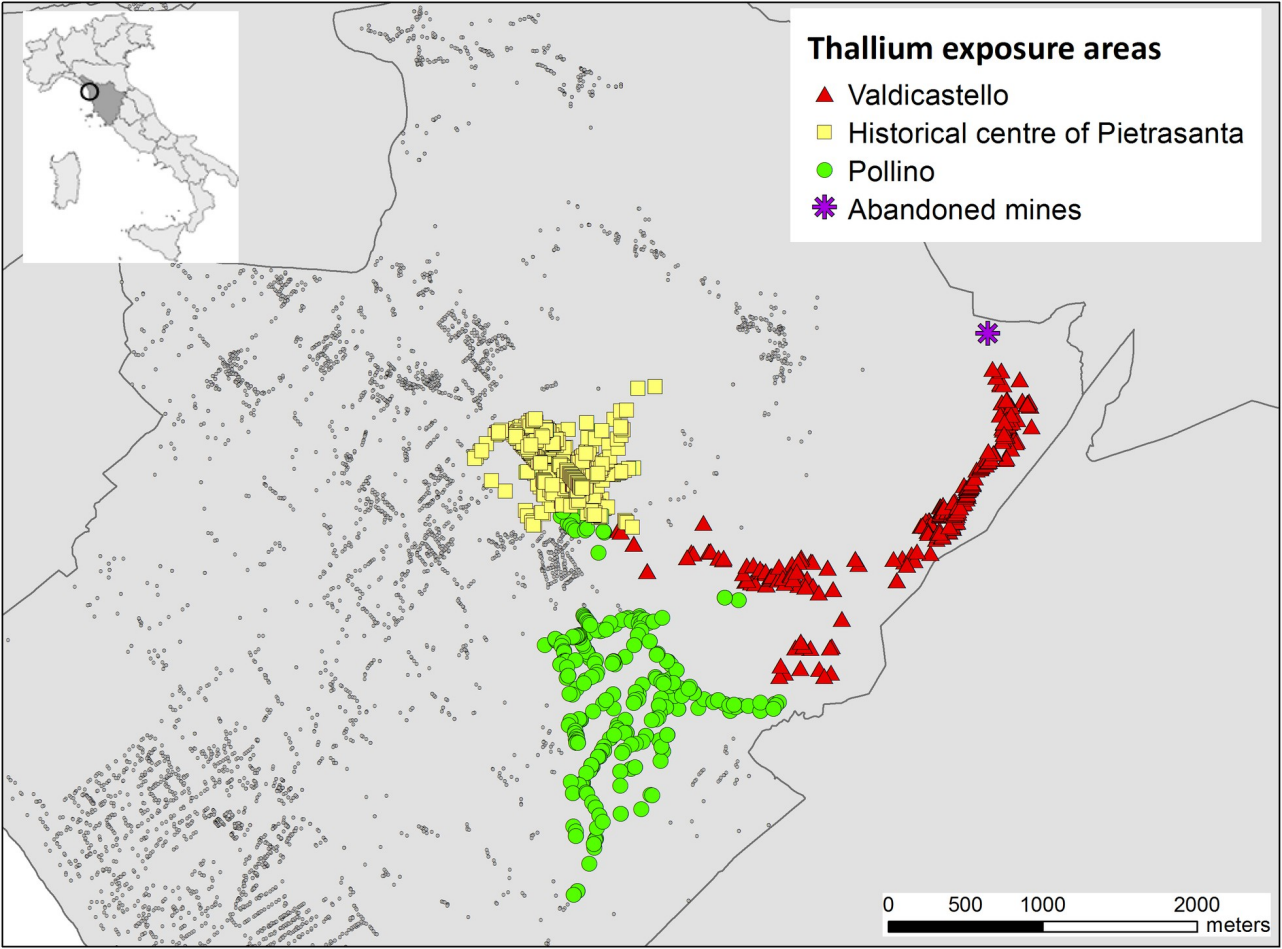
Map of the thallium contaminated areas. In red the Valdicastello area (concentration of TI in water up to 79.5 μg/L), in yellow the centre of Pietrasanta (concentration of TI in the water up to 6.80 μg/L), in green the Pollino area (concentration of TI in the water up to 8.40 μg/L), in grey the area not affected by the pollution (concentration of TI in the water up to 2.0 μg/L). Data source: Tuscany Region—datasets “ambiti_amministrativi.zip and iternet.zip” released under the Creative Commons Attribution 4.0 license.

Fig 1 shows a map of the areas affected by Tl contamination and by the Do Not Drink orders. In Valdicastello area concentrations of TI in the tap water were up to 79.5 μg/L, in the old town centre of Pietrasanta up to 6.80 μg/L, and in the Pollino area up to 8.40 μg/L. According to the numerous data of water samples taken by the supplier and by the Local Health Authority (around 4,800 samples in the two years following the contamination), the area of Valdicastello was divided into three further zones: Valdicastello upper (concentration of TI in tap water up to 79.5 μg/L), Valdicastello middle (concentration of TI in tap water up to 55.4 μg/L) and Valdicastello lower (concentration of TI in tap water up to 43.8 μg/L).

### Urinary thallium concentrations

A total of 2,154 urine samples were analysed. Table 1 shows the size of the urine samples for each of the six homogeneous groups, the size of the samples excluded for creatinine values outside the acceptability range and the number of outliers. Table 1 also shows the descriptive statistics of the various groups of urine samples expressed in μg/L and in μg/g creatinine and the number and the percentage of samples that exceeds the 95th percentile of reference values of the Italian non-smoker population, 0.759 μg/L [1], of the total US population sampled over the period 2015–2016, 0.435 μg/L [23] and of the population of Belgium [25] and [9] France, 0.5 μg/L.

**Table 1:** Descriptive statistics of Tl urinary concentrations by groups.

		Group 1^1^	Group 2^2^	Group 3^3^	Group 4^4^	Group 5^5^	Group 6^6^
**N. (%) of the total samples**		636 (29.5)	696 (32.3)	334 (15.5)	155 (7.2)	118 (5.5)	215 (10.0)
**N. (%) creat. in the range^7^**		571 (89.8)	671 (96.4)	303 (90.7)	143 (92.2)	108 (91.5)	204 (94.5)
**N. outliers**		75	17	2	1	38	2
**AM^8^±SD^9^**	μg/L	0.670±0.680	0.434±1.14	0.211±0.182	0.288±0.221	0.231±0.214	0.295±0.772
	μg/g creat.	0.751±0.808	0.396±0.574	0.226±0.168	0.303±0.225	0.269±0.331	0.258±0.600
**GM^10^ (95%CI)**	μg/L	0.467	0.300	0.171	0.228	0.186	0.198
		(0.434–0.502)	(0.282–0.317)	(0.159–0.183)	(0.204–0.255)	(0.165–0.208)	(0.179–0.218)
	μg/g creat.	0.522	0.296	0.187	0.241	0.203	0.173
		(0.487–0.559)	(0.281–0.312)	(0.174–0.200)	(0.215–0.270)	(0.179–0.229)	(0.157–0.190)
**Min-Max**	μg/L	0.010–8.96	0.010–27.9	0.029–1.86	0.029–1.56	0.030–1.80	0.033–10.9
	μg/g creat.	0.058–11.1	0.048–11.6	0.057–1.20	0.029–1.18	0.050–2.88	0.017–7.49
**P5**	μg/L	0.125	0.091	0.064	0.068	0.084	0.066
	μg/g creat.	0.140	0.112	0.082	0.081	0.087	0.077
**P25**	μg/L	0.274	0.193	0.114	0.144	0.134	0.135
	μg/g creat.	0.289	0.185	0.123	0.157	0.129	0.117
**P50 (95%CI)**	μg/L	0.443	0.302	0.240	0.169	0.171	0.200
		(0.412–0.495)	(0.283–0.317)	(0.212–0.276)	(0.156–0.182)	(0.158–0.189)	(0.179–0.217)
	μg/g creat.	0.509	0.283	0.172	0.240	0.185	0.159
		(0.461–0.547)	(0.267–0.299)	(0.161–0.191)	(0.223–0.279)	(0.164–0.201)	(0.149–0.179)
**P75**	μg/L	0.836	0.468	0.247	0.361	0.257	0.289
	μg/g creat.	0.915	0.445	0.257	0.378	0.294	0.242
**P95**	μg/L	1.89	0.952	0.475	0.641	0.527	0.563
	μg/g creat.	2.19	0.937	0.607	0.797	0.577	0.454
**N (%) > 0.759 μg/L^11^**	174 (30.5)	60 (8.9)	4 (1.3)	5 (3.5)	3 (2.8)	6 (2.9)	
**N (%) > 0.435 μg/L^12^**	294 (51.5)	191 (28.5)	17 (5.6)	23 (16.1)	7 (6.5)	22 (10.8)	
**N (%) > 0.50 μg/L^13^**	258 (45.2)	145 (21.6)	14 (4.6)	19 (13.3)	7 (6.5)	15 (7.4)	

^1^Volunteers A;

^2^Volunteers B;

^3^Active invitation Pietrasanta town centre;

^4^Pollino A;

^5^Pollino B;

^6^Controls;

^7^samples with creatinine concentrations within the WHO guidelines >0.3 g/L and <3.0 g/L [34];

^8^arithmetic mean;

^9^standard deviation;

^10^geometric mean;

^11^95P Italian population [1];

^12^P95 US population [23];

^13^P95 Belgium [25] and France [9] population.

The analysis of variance, performed on the logarithms of the data, showed a significant difference between the six groups (p<0.0001): as expected, group A of the volunteers had statistically higher values of urinary TI than all the other groups; the sample group B of the volunteers also showed statistically higher values than the other groups (groups 3–6). The samples A from Pollino had concentrations statistically higher than the samples B from Pollino (p = 0.0007) and the active invitation group from Pietrasanta (group 3) even had statistically lower concentrations than the control group (group 6).

Analysing the groups of volunteers separately, we found a statistically significant reduction between samples A and samples B (p<0.0001). In group 1, 30.5% of samples exceeded the 95th percentile of the reference Italian population; this percentage decreased to 8.9% after the end of the exposure (group 2).

**Fig 2:**
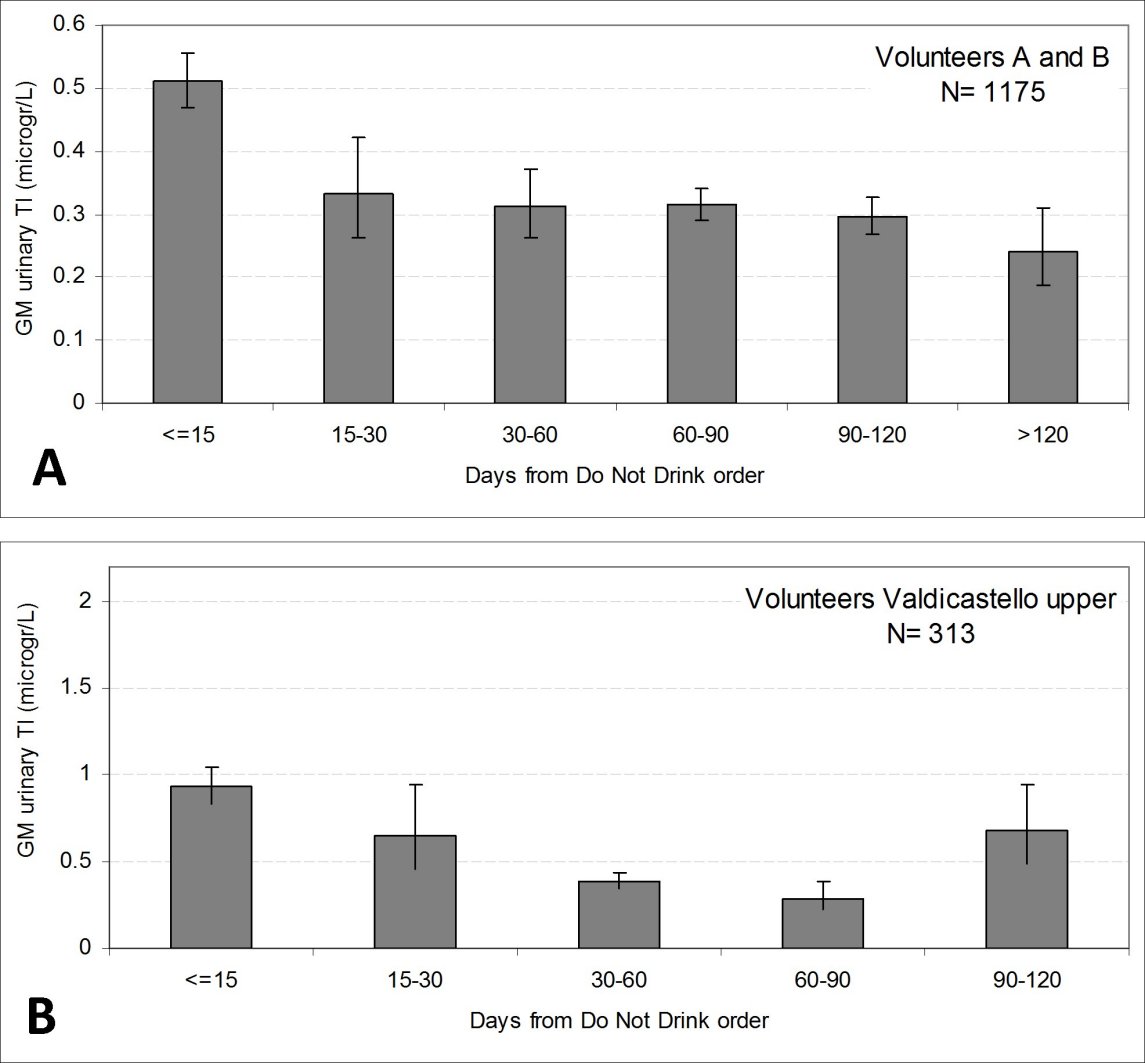
Temporal pattern of Tl urinary excretion: Geometric means (95% confidence intervals) by days from the Do Not Drink order. (A): groups of volunteers A and B. (B): Valdicastello upper volunteers.

Fig 2 shows the reduction of Tl urinary levels on the basis of the increase in number of days elapsed between the Do Not Drink order and the collection of the samples. The temporal trends are referred to the groups of volunteers A and B (Fig 2A), and to the Valdicastello upper volunteers (Fig 2B).

In the group of volunteers A and B, urinary Tl concentrations decreased approximately by 35% in the first period (15–30 days after the order), suggesting a fast release within the first month after the end of exposure, and a very slow release in the following months. In the subset of Valdicastello upper samples, the decrease is slower, even though the number of samples in the second and in the last period are very low, 5 and 4 samples respectively.

The results of the univariate regression models, restricted to the group of volunteers (groups 1 and 2), are shown in Table 2.

**Table 2:** Results of the univariate regression models for the groups of volunteers.

	Group 1 –Volunteers A				Group 2 –Volunteers B			
	N	β^1^	95%CI	p	N	β^1^	95%CI	p
**Sex**								
Males	271	Ref.			301	Ref.		
Females	300	0.968	0.836–1.119	0.657	370	0.825	1.042–0.202	0.927
**Age groups (years)**								
0–18	146	Ref.			171	Ref.		
19–40	118	1.150	0.927–1.425	0.203	143	1.404	1.188–1.660	<0.001
45–55	149	1.278	1.045–1.564	0.017	168	1.492	1.271–1.751	<0.001
>55	158	1.160	0.950–1.415	0.144	189	1.259	1.078–1.471	0.004
**Residence area**								
Pietrasanta	129	Ref.			151	Ref.		
Valdicastello lower	70	1.044	0.836–1.31	0.703	93	1.037	0.855–1.256	0.714
Valdicastello middle	208	1.584	1.339–1.87	0.001	251	1.385	1.192–1.609	<0.001
Valdicastello upper	164	3.133	2.626–3.74	<0.001	176	1.598	1.359–1.878	<0.001
**Duration of residence**								
<10 years	175	Ref.			214	Ref.		
>10 years	396	1.113	0.951–1.304	0.181	457	1.163	1.027–1.316	0.017
**Education**								
Low	169	Ref.			191	Ref.		
Medium	149	1.304	1.076–1.581	0.007	180	1.319	1.131–1.539	<0.001
High	206	1.021	0.854–1.220	0.820	241	1.268	1.098–1.464	0.001
**BMI**								
Normal	280	Ref.			324	Ref.		
Overweight/Obesity	219	1.203	1.032–1.403	0.019	259	1.115	0.983–1.266	0.090
**Smoking habits**								
Never smoker	304	Ref.			356	Ref.		
Ex-smoker	120	1.054	0.874–1.270	0.584	136	1.081	0.928–1.259	0.316
Smoker	96	1.110	0.906–1.359	0.314	115	1.188	1.010–1.398	0.037
**Consumption of drinking water from the aqueduct**	545	1.451	1.251–1.682	<0.001	640	1.100	0.975–1.241	0.121
**Consumption of locally produced food**	571	1.199	1.037–1.386	0.014	671	1.182	1.054–1.327	0.004
**Consumption of locally produced vegetables or fruits**	522	1.220	1.044–1.425	0.012	610	1.096	0.968–1.240	0.148
**Usual consumption of wine**	571	1.125	0.962–1.316	0.140	671	1.267	1.118–1.436	<0.001
**Usual consumption of tea**	440	1.108	0.928–1.323	0.257	512	1.122	0.974–1.293	0.111
**Usual consumption of coffee**	571	1.157	0.995–1.346	0.069	671	1.241	1.102–1.398	<0.001
**Usual consumption of vegetables^2^**	535	0.946	0.771–1.159	0.590	626	1.066	0.902–1.259	0.453
**Usual consumption of fish^3^**	529	1.181	0.967–1.443	0.103	625	1.106	0.947–1.292	0.204
**Tattoos**	516	0.938	0.754–1.167	0.564	604	0.901	0.759–1.068	0.229
**Piercings**	510	1.073	0.914–1.260	0.389	601	0.985	0.868–1.117	0.813

^1^exponential of the coefficients of the regression models using ln-transformed Tl concentration as dependent variable;

^2^more than 5–6 times per week;

^3^more than 1–2 times per week.

A clear geographical gradient was observed both in samples A and B. In samples A the residence in Valdicastello upper was strongly associated with increasing Tl urinary levels, resulting in an increase of 213% on the geometric mean with respect to the reference group. In samples A the geometric means were 0.887 μg/L in Valdicastello upper, 0.448 μg/L in Valdicastello middle, 0.296 μg/L in Valdicastello lower, and 0.283 μg/L in the historical centre of Pietrasanta. Drinking tap water was significantly associated with increasing Tl levels in group 1 of volunteers A (+45%, 95%CI: 25–68%), but not in group 2. In both the groups, the associations with the consumption of locally produced food, vegetables or fruits in particular, were statistically significant (+22% in group 1 and +10% in group 2). In the group of volunteers B, for which the contribution of the exposure to TI resulting from contamination of drinking water was much lower than in the group A, the association with smoking was statistically significant (+19% in smokers). In group 2 other associations were observed with age groups, residence for more than 10 years, education, and the usual consumption of wine and coffee. In both groups the age range with the highest urinary Tl levels was 45–55 years. Gender did not result in significant association with increasing Tl levels in both groups of volunteers.

The univariate regression models, applied to the groups of samples collected by active invitation in Pietrasanta town centre and in Pollino district, reported significant associations with gender (in females 23% decrease in geometric mean with respect to men), usual consumption of wine (+24%), consumption of locally produced vegetables or fruits (+18%), and piercing (+25%). In the control group urinary Tl levels were significantly associated with gender (females +10%), usual consumption of wine (+13%) and tea (+10%).

In the multivariate models (Table 3), including the variables resulting in the highest R^**2**^ in the group 1 of volunteers A, age group, the area of residence, drinking tap water, the consumption of locally produced vegetables or fruits, the usual consumption of fish as well as the ln-transformed creatinine remained significantly and positively associated with Tl urinary concentration (R^**2**^ = 0.46). In the group 2 of volunteers B, age group, area of residence and urinary creatinine remained significantly associated with Tl urinary levels (R^**2**^ = 0.33), while other predictors, such as smoking habits and education resulted non-significantly associated.

**Table 3:** Results of the multivariate regression models for the groups of volunteers.

	Group 1 –Volunteers A				Group 2 –Volunteers B			
	β^1^	95%CI	p	R^2^	β^1^	95%CI	p	R^2^
**Sex**				0.46				0.33
Males	Ref				Ref			
Females	1.003	0.871–1.155	0.966		1.066	0.952–1.194	0.265	
**Age groups (years)**								
0–18	Ref.				Ref			
19–40	0.902	0.723–1.126	0.362		1.210	0.953–1.536	0.117	
45–55	1.111	0.897–1.375	0.334		1.407	1.109–1.784	0.005	
>55	1.297	1.035–1.625	0.024		1.464	1.182–1.814	<0.001	
**Residence area**								
Pietrasanta	Ref				Ref			
Valdicastello lower	1.050	0.801–1.375	0.725		0.852	0.698–1.041	0.119	
Valdicastello middle	1.342	1.095–1.645	0.005		1.258	1.070–1.478	0.005	
Valdicastello upper	2.588	2.098–3.192	<0.001		1.462	1.236–1.728	<0.001	
**BMI**								
Normal	Ref.							
Overweight/Obesity	1.090	0.948–1.254	0.223		n.d.	n.d.	n.d.	
**Consumption of drinking water from the aqueduct**	1.249	1.088–1.433	0.002		n.d.	n.d.	n.d.	
**Consumption of locally produced vegetables or fruits**	1.120	0.076–1.670	0.095		1.104	0.983–1.239	0.093	
**Usual consumption of fish**	1.188	1.000–1.411	0.050		n.d.	n.d.	n.d.	
**Urinary creatinine (ln)**	2.046	1.838–2.279	<0.001		2.021	1.826–2.236	<0.001	
**Smoking habits**								
Never smoker					Ref			
Ex-smoker	n.d.	n.d.	n.d.		0.947	0.822–1.092	0.457	
Smoker	n.d.	n.d.	n.d.		0.890	0.764–1.037	0.138	
**Education**								
Low					Ref			
Medium	n.d.	n.d.	n.d.		0.961	0.803–1.150	0.668	
High	n.d.	n.d.	n.d.		0.926	0.767–1.118	0.426	
**Usual consumption of wine**	n.d.	n.d.	n.d.		1.096	0.970–1.238	0.140	

^1^exponential of the coefficients of the regression models using ln-transformed Tl concentration as dependent variable.

### Hair thallium concentrations

A total of 254 hair samples were analysed. Table 4 shows the descriptive statistics of the hair samples for each of the four groups.

**Table 4:** Descriptive statistics of hair Tl concentrations by groups (ng/g).

Group	N.	AM±SD	GM (95% CI)	Min-Max	P5	P25	P50 (95% CI)	P75	P95
**Group 1^1^**	14	1.31±1.64	0.72 (0.38–1.4)	0.10–6.3	0.1	0.31	0.71 (0.30–1.75)	1.67	6.29
**Group 2^2^**	138	1.69±3.23	0.74 (0.60–0.90)	0.10–28.3	0.14	0.28	0.59 (0.49–0.84)	1.67	6.90
**Group 3^3^**	43	1.88±3.00	0.87 (0.60–1.3)	0.11–15.2	0.18	0.36	0.85 (0.42–1.30)	2.13	6.02
**Group 4^4^**	59	0.50±0.42	0.36 (0.29–0.45)	0.10–1.75	0.10	0.21	0.36 (0.28–0.50)	0.67	1.52

^**1**^Volunteers;

^**2**^Active invitation Pietrasanta;

^**3**^Active invitation Pollino;

^**4**^Controls.

The analysis of variance, performed on the ln-transformed data, shows that the groups of people living in the three main exposed areas (Valdicastello, Pietrasanta old town centre and Pollino) have levels of TI in hair statistically higher than the controls (p = 0.0001). However there are no significant differences among the three groups of the more exposed people.

With regard to the associations with the basic characteristics of the subjects, statistically significant differences were reported in the elderly (+95%) and in people regularly consuming locally grown vegetables or fruits (+90%).

In the group of samples from Pietrasanta town centre several statistical tests were used to evaluate the correlation between the levels of Tl in the urine and in the hair. Spearman and Kendall tests reported significant positive correlation between levels in urine and hair (p = 0.018 for both tests).

### Self-reported health effects

Table 5 shows the results obtained from the multivariate logistic regression models between urinary, or hair, Tl concentrations and the prevalence of various self-reported symptoms and health effects. The results are referred to the group 1 of volunteers A, i.e. the most exposed subjects, with regard to urine, and to the whole sample (254 samples) with regard to hair.

**Table 5:** Associations between urinary and hair thallium (Tl) concentrations and self-reported symptoms. Odds Ratio (OR) and 95% confidence intervals (95%CI).

	Urinary Tl Group 1 –Volunteers A				Hair Tl All samples			
	N	OR^1^	95%CI	p	N	OR^2^	95%CI	p
**Alterations of skin, hair or nails**								
Dermatitis	203	1.09	0.90–1.31	0.385	83	1.04	0.86–1.27	0.684
Loss of hair, alopecia	153	1.05	0.86–1.27	0.654	53	0.97	0.77–1.21	0.770
White transverse striations of finger nails	151	0.92	0.75–1.12	0.397	78	1.08	0.88–1.32	0.449
**Oral cavity and gastro-intestinal dysfunctions**								
Glossitis, stomatitis	98	0.98	0.78–1.22	0.853	42	0.97	0.76–1.23	0.810
Nausea	183	0.95	0.78–1.14	0.563	55	0.99	0.79–1.25	0.945
Anorexia, loss of weight	38	1.41	0.97–2.05	0.099	14	1.34	0.84–2.13	0.221
Obstipation, diarrhea	345	1.06	0.88–1.27	0.539	136	1.00	0.83–1.20	0.996
**Neurologic and subjective symptoms**								
Visual disorders	80	1.13	0.88–1.46	0.325	25	1.23	0.91–1.66	0.182
Hyperaesthesia at the lower extremities	80	1.28	0.97–1.68	0.097	22	0.79	0.57–1.10	0.165
Tachycardia, extrasystoles	136	1.01	0.82–1.24	0.931	49	0.83	0.65–1.07	0.146
Sleep disturbance	202	1.20	0.99–1.46	0.064	82	1.27	1.04–1.55	0.019
Other signs of polyneuropathy or psychasthenia	33	0.80	0.59–1.09	0.158	12	0.99	0.66–1.49	0.964

^1^Odds Ratio from multivariate logistic regression models, adjusting for gender, age, area of residence, education, ln-transformed urinary creatinine;

^2^Odds Ratio from multivariate logistic regression models, adjusting for gender, age, smoking habits and education.

The increasing in Tl urinary and hair concentrations were not associated with self-reported symptoms potentially related to Tl exposure, except for the disturbance of sleep, which resulted associated with both urinary (+20%, 95%CI: 0–46%) and hair (+27%, 95%CI: 4–55%) levels. With regard to urinary Tl levels, no associations were found with self-reported symptoms when the models were run in the other sample groups (groups 2–6, data not shown).

## Discussion

Thallium contamination of the water distribution system in some areas of the municipality of Pietrasanta, Italy was an extremely rare event. A similar case occurred in the '60s in Southwest China in a region rich in sulphide minerals containing this element released through natural weathering processes and/or mining activities [18]. The population was exposed to very high levels of Tl, resulting in severe health effects [16].

The biomonitoring survey conducted in Pietrasanta was performed using different methods for the recruitment of the participants. The first samples (group 1 of volunteers A) were collected on a voluntary basis and in a rather short time, in order to quickly evaluate the presence of high levels of exposure and, if necessary, to decide therapeutic treatment. The second sample set was collected with two main objectives. The first was to evaluate the urinary Tl levels after the end of the main exposure, when the Do Not Drink order was established and emergency measures were implemented to lower the Tl levels in drinking water. The second was to evaluate the impact of the contamination in the other areas affected by the orders, but were situated farther away from the contamination source. Thus, due to the geographical and temporal pattern of the collected samples, six homogenous groups were identified and separately analysed. The group 1 of volunteers A, the more exposed subjects, reported Tl urinary levels higher than reference values for the general populations reported in national surveys. The geometric mean and the 95th percentile in Group 1 were 0.467 μg/L and 1.89 μg/L respectively, and the corresponding reference values are 0.203 μg/L and 0.759 μg/L in the Italian population [1], 0.153 μg/L and 0.435 μg/L in the US national survey [23], 0.21 μg/L and 0.48 μg/L in a general adult population of Northern France [9], 0.168 and 0.500 μg/L in a adult population of Belgium [25]. The 31% of samples of volunteers A exceeded the 95th percentile of the Italian reference population. Similar values were reported in a study conducted in the same area: in 150 urine samples Tl levels ranged from 0.046 to 5.44 μg/L, with geometric mean 0.55 μg/L and 95th percentile 1.88 μg/L [31]. However the urinary Tl levels measured in the group of volunteers A were lower than the values reported in other populations, who underwent environmental Tl exposure [14–16, 19, 20, 24].

A geographical gradient in urinary Tl levels was observed in the group of volunteers A, a decrease was evident with increasing distance from the source of contamination, closely related to the Tl levels measured in tap water samples. People living in Valdicatello upper showed the highest urinary Tl levels: the geometric means ranged from 0.887 μg/L in Valdicastello upper area to 0.283 μg/L in the historical centre of Pietrasanta. Emergency measures were quickly implemented to lower the levels of Tl in the water distribution system. Orders banning the usage of water were imposed and control measures, including extensive system flushing, complete replacement of some stretches of steel pipes, and innovative technique to clean the remaining contaminated pipes, were effected to bring back Tl levels in drinking water to guidelines values, 0.2 μg/L according to EPA standard limits [12]. These emergency measures significantly reduced Tl urinary levels: geometric means passed from 0.467 μg/L in samples A to 0.300 μg/L in samples B of volunteers. The percentage of samples exceeding the 95th percentile of the Italian reference population passed from 30.5% to 8.9%. A reduction was also observed in the samples collected in the Pollino area, before and after the Do Not Drink order. Even though urinary levels in the Pollino area were much lower than those observed in the group of the Valdicatello area, the geometric means passed from 0.228 μg/L in group 4 to 0.186 μg/L in group 5. The samples collected in the historical centre of Pietrasanta, several weeks after the end of the main exposure from the contaminated drinking water system, showed urinary Tl levels even lower than in the control groups.

The analyses of samples stratified by the habit of drinking tap water confirmed the time frame of the different sample groups. Urinary Tl levels resulted significantly associated with drinking tap water only in volunteer group A. The lack of significance in the group of volunteer group B is clearly associated with the time of sample collection. The significant associations reported with the consumption of locally produced food for both samples A and B suggest that this way of exposure can be prolonged even for very long periods. Other predictors such as smoking habits, the length of time lived in the contaminated areas, the usual consumption of wine and age resulted associated with urinary Tl levels only in the samples of group B, for which the main source of intake of Tl was greatly reduced due to the ban on the use of contaminated water. Thus, in the group A of volunteers the impact of the exposure to the contaminated drinking water was so high as to mask the effects of other potential predictors, which were instead highlighted in the other group of samples.

The availability of samples collected at different time periods allowed us to evaluate the pattern of Tl decay. We observed a fast reduction of the urinary Tl levels in the first month after the Do Not Drink order, which is probably due to the release from soft tissue (i.e. muscle, kidney, liver), while release from the bone could be slower [36].

Hair samples are generally used to measure historic exposure to metals and, based on monthly growth rate, approximately one centimetre, they reflect exposure to the elements over months or even years [37]. Over time the concentration in the hair increases and this matrix can contain most of the body’s load of TI, providing an important additional route of slow elimination from the body [5, 37]. Hair samples in this study were collected in the different exposure areas, and geometric means were higher than in control groups: 0.73 ng/g in Valdicastello, 0.74 ng/g in the historical centre of Pietrasanta, 0.87 ng/g in Pollino, and 0.36 ng/g in controls. The samples collected in Valdicastello were very few (14 hair samples) but other authors reported a geometric mean of 15 ng/g in 318 hair samples collected in this area [31]. Few other studies reported Tl concentration in hair samples: Batista et al. found a geometric mean of 0.3 ng/g in unexposed population [30] and Violante et al. an arithmetic mean of 1 ng/g in 92 children (9–10 years old) living nearby a thermoelectric power plant [32]. The high variability observed in the group of exposed people is likely linked to the characteristics of this biological matrix: the difference in length of the hair involves taking the different history of exposure into account.

In our study the concentrations of Tl in urine and hair were confirmed as being useful indicators of internal exposure. Whereas hair is useful for the assessment of chronic or long-term exposure, urinary excretion of metals represents the amount removed from the body, related to recent exposure. Thallium elimination through urine takes place over a period of 10–30 days [5, 38] with a physiological half-life of 12.5 days [38]. The debate about the validity of the quantitative results from hair analysis and their interpretation is still open, because of some unresolved questions such as the influences of contamination by exogenous materials such as soil, dust, and water, or cosmetic treatment, and possible genetic differences [39]. Despite these critical issues, we observed a significant correlation between levels found in urine and hair for the group of people living in Pietrasanta. The significant correlation between urine and hair can be connected with the time the biological samples were collected, with possible exposure that was not one-off but instead continuous, including through food, and with the different degree of elimination of the metal through the two routes: it has been documented that renal excretion can reach to around 73%, against the elimination through hair and skin of approximately 19.5% [5].

As in other biomonitoring studies, we reported urinary Tl levels as raw concentrations and corrected by urinary creatinine. According to WHO guidelines [34], too diluted urine samples (creatinine concentrations <0.3 g/L) may compromise the detection of low levels of toxicants; in the same way samples with very high creatinine concentrations (>3.0 g/L) indicate dehydration, which could have changed the kidney’s secretion, excretion, and/or reabsorption of the target chemical. For these reasons the creatinine-adjusted concentration is often preferred to raw concentrations. However, urinary creatinine levels are dramatically different among various demographic groups. Thus in multiple regression analysis a different approach was proposed to take into account the impact this heterogeneity could have on results [35]. The authors recommend the inclusion of the analyte concentration (unadjusted for creatinine) in the multiple regression analysis and to add urinary creatinine as a separate independent variable. We applied this method in our multiple regression models, consequently the urinary Tl concentration is appropriately adjusted for urinary creatinine and the statistical significance of other variables in the model (e.g., age, sex) emerges to be independent from the effects of urinary creatinine concentration.

Regarding health effects, few studies have been conducted in populations chronically exposed to low levels of Tl [19–22]. In our study increasing levels of Tl in urine and hair resulted in an association with increased risk of sleep disturbance. No positive correlation was found between Tl levels in hair and urine and the prevalence of skin alterations, hair-loss and gastro-intestinal dysfunctions. The association between urinary and hair Tl concentrations and self-reported symptoms allowed to confirm 5 μg/L as the level below which toxic effects are not probable [5]. Brockhaus et al. [19] reported similar findings in a cross-sectional survey conducted in a population living around a Tl emitting cement plant in a small city in North-West Germany. Authors observed a clear exposure-response relationship between urinary and hair Tl levels and the prevalence of sleep disorders, tiredness, weakness, nervousness, headache, and other psychic alterations. However, such self-reported symptoms have to be interpreted carefully because of potential “recall bias” and “recall enhancement bias”, especially in the groups of volunteers highly involved in the emergency situation.

The strength of this work is the very large number of Tl data in urine samples collected in a limited area; this allows to clarify the levels of excretion associated with situations of pollution of the water destined for human consumption. If the associations with the consumption of locally produced food was possible through the questionnaire information, however, some limitations are evident and associated with the lack of specific data on the concentration of airborne thallium and in foods actually consumed by the population. The real weakness of this study is the limited number of hair samples analysed, especially for the most exposed group. This makes it impossible to perform a thorough statistical inference on this type of biological matrix, for which the number of studies is still limited. Another study limitation is the potential misclassification of the included predictors, as all the explanatory variables (smoking, education, dietary habits) were self-reported. Although recall bias is a major issue in studies that have self reporting, internal and external consistence of our results do not suggest any relevant impact.

## Conclusions

This study assessed Tl exposure in a population residing in some areas of the municipality of Pietrasanta, Italy where contamination from this metal occurred in water for human consumption. The analysis of a substantial number of urine samples, which differed among other things, on the time of collection and the geographical area of residence of population, allowed to highlight levels of excretion of Tl significantly higher than the reference values of the Italian general population and an appropriately selected control group. The analysis of hair, although performed on a smaller number of samples, confirmed an accumulation of Tl in the organism for the groups most exposed. An in-depth statistical inference of urine data has highlighted the significant influence of some factors such as age, residence and duration of residence in areas with greater contamination, the level of education, the habit of smoking tobacco, the consumption of drinking water from the aqueduct and locally produced food, wine and coffee.

The Tl contamination of the water supply network in the town of Pietrasanta was an extraordinary experience for all people involved: citizens, administrators, health authorities, water supplier, technicians and researchers. In a climate of population alarm and strong media pressure, thanks to a synergistic and multidisciplinary approach, the emergency was dealt with appropriately, restoring acceptable levels of TI in water. The timely collection of urine samples, made possible by the voluntary actions of large numbers of local people, enabled the very rapid exclusion of serious risks to the health of the population. This event has, however, also revealed the limits of the old structure of management and protection of drinking water, which was later finally superseded by the new Directive 2015/1787/EC. Indeed, a new holistic approach, the Water Safety Plans (WSP) model, is encouraged: it pursues a global system of risk assessment and risk management extended to the whole water supply chain, from collection to end users [40].

To date, this study is the most extensive campaign of human biomonitoring to assess exposure to TI. It provides a significant contribution to the state of knowledge, still very poor in terms of levels and determinants of exposure to Tl for the general population and on the health effects of low-level chronic exposure.
